# Massive citizen science sampling and integrated taxonomic approach unravel Danish cryptogam-dwelling tardigrade fauna

**DOI:** 10.1186/s12983-024-00547-x

**Published:** 2024-10-21

**Authors:** Piotr Gąsiorek, Martin V. Sørensen, Marie Rathcke Lillemark, Frederik Leerhøi, Anders P. Tøttrup

**Affiliations:** 1 Natural History Museum of Denmark, University of Copenhagen, Copenhagen, Denmark; 2 Department of Invertebrate Evolution, Faculty of Biology, Jagiellonian University, Kraków, Poland

**Keywords:** Citizen science, Cosmopolitan, DNA barcoding, Faunistics, Morphology, Palaearctic, Rare species, Species checklist

## Abstract

Tardigrade diversity and distribution are enigmatic in most parts of the globe, and only some European countries can boast of a relatively well-studied water bear fauna. However, even these suffer from the lack of genetic data, which would substantiate faunistic data and make biogeographic comparisons easier. Denmark has never been intensively and systematically researched in this regard, thus a citizen science sampling of cryptogams (mosses, liverworts, and lichens) was launched in spring 2023, aiming at a comprehensive biodiversity survey across this insular country. Nearly 700 samples were selected out of 8.000 sent to NHMD, based on the quality of samples, representativeness of various regions of Denmark, and the type of substrate to allow unravelling of potential ecological associations between tardigrades and cryptogams. Importantly, a large fraction of morphological identifications was backed up by DNA barcode data based on ITS-2 (1001 sequences), and in some cases also on COI (93 sequences) and ITS-1 (22 sequences) molecular markers, which are recognised DNA fragments used in species delimitation. We quadruple the number of known Danish limno-terrestrial tardigrade species (55 spp. reported in this paper *vs*. 14 spp. reported in literature so far, most of which were contentious due to the insufficient knowledge on tardigrade taxonomy), demonstrating the power of integrative taxonomy. No fewer than nine spp. are new to science. This is the first case where tardigrade fauna of an entire country is examined both from morphological and DNA barcoding data perspective.

**Supplementary Information:**

The online version contains supplementary material available at 10.1186/s12983-024-00547-x.

## Introduction

Out of the seven deficiencies that torment biologists exploring biodiversity [1], the most primeval are the lack of knowledge on taxonomy (Linnean shortfall) and biogeography of organisms (Wallacean shortfall). While the first is currently being addressed with comprehensive sampling and phylogenomic data even for microscopic animals [2], the latter is more grave with a decreasing body size of studied organismal group [3]. Tardigrades, the closest relatives of arthropods and onychophorans [4, 5], represent meiofauna (microfauna) both in marine and terrestrial habitats. Both shortfalls are utterly timely in their case: tardigrade classification undergoes revolution thanks to the integrative approach, converging classical light microscopy, scanning electron microscopy, karyotyping, and DNA barcoding [6, 7] into reliable species hypotheses and higher rank systematics. The process of defining species distributions and biogeography of tardigrades suffer from scanty and biased sampling, but most recent studies indicate biogeographic structuring, in contrast with the previously purported prevalent cosmopolitanism [8, 9]. This translates into the need of further biodiversity surveys supported by DNA evidence, which increases objectivity, enhances comparability between various studies, and thus reduces the risk of establishing synonyms [10, 11].

The Danish tardigrade fauna has been meagrely researched, with the current species count standing at 18 spp. (four marine and 14 limno-terrestrial, see Table 1 for details). This stays in a stark contrast to the nearby Sweden ([12]: 101 spp.; a long history of research since the times of Thulin [13, 14]) and Norway ([15]: 146 spp.). Only the latest survey employed a V4 region of the 18S rRNA marker in environmental DNA metabarcoding of Danish soil samples [16] to uncover multiple eutardigrade lineages. Pust et al. [16] revealed that Danish fauna embraces 96 (!) molecular operational taxonomic units (MOTUs), which could correspond to species (however, a species delimitation based on 18S rRNA chiefly underestimates true α-diversity, so some MOTUs may represent more spp.). An important achievement of this study was the discovery of presence of virtually all (11) eutardigrade families, which could be suspected of occurring in Denmark. The same goes for several genera, which otherwise would be particularly difficult to extract from samples via traditional laboratory methods (*Bertolanius*, *Eohypsibius*, *Eremobiotus*, *Hexapodibius*, *Microhypsibius*, *Mixibius*) due to rareness. Hence, we consider the list of MOTUs provided by Pust et al. [16] to constitute a backbone for modern faunistic research on Danish tardigrades, which must be corroborated by both morphological and molecular evidence.

**Table 1:** List of Danish Tardigrada recorded prior to this study (synonyms excluded)

Family	Species and authority	Source	Status in Denmark
*Marine*			
Batillipedidae	1. *Batillipes mirus* Richters, 1909	[65]	Valid
Echiniscoididae	2. *Echiniscoides sigismundi* (M. Schultze, 1865)	[65, 76]	Valid*
Halobiotidae	3. *Halobiotus crispae* Kristensen, 1982	[77, 78]	Valid
	4. *Halobiotus geddesi* (Hallas, 1971)	[79]	Uncertain**
*Limno-terrestrial*			
Echiniscidae	1. *Echiniscus testudo* (Doyère, 1840)	[65, 80, 81]	Valid
	2. *Pseudechiniscus suillus* (Ehrenberg, 1853)	[65]	Questionable
Milnesiidae	3. *Milnesium tardigradum* Doyère, 1840	[65, 77]	Questionable
Hypsibiidae	4. *Adropion scoticum* (Murray, 1905)	[65, 67]	Questionable
	5. *Degmion oculatum* (Murray, 1906)	[67]	Questionable
	6. *Diphascon alpinum* Murray, 1906	[65, 67]	*nomen dubium*
	7. *Diphascon stappersi* Richters, 1911	[67]	Questionable
	8. *Hypsibius dujardini* (Doyère, 1840)	[65, 67]	Questionable
	9. *Pilatobius bullatus* (Murray, 1905)	[67]	Questionable
Ramazzottiidae	10 *Ramazzottius oberhaeuseri* (Doyère, 1840)	[65, 77]	Questionable
Doryphoribiidae	11. *Grevenius granulifer* (Thulin, 1928)	[78]	Valid
Isohypsibiidae	12. *Isohypsibius prosostomus* Thulin, 1928	[67, 78]	Questionable
Macrobiotidae	13. *Macrobiotus hufelandi* C.A.S. Schultze, 1834	[65, 67]	Questionable
	14. *Mesobiotus harmsworthi* (Murray, 1907)	[65, 67]	Questionable
	15. *Minibiotus intermedius* (Plate, 1888)	[65]	Questionable
Murrayidae	16. *Dactylobiotus macronyx* (Dujardin, 1851)	[65]	*nomen dubium*

A species’ presence in Denmark was considered questionable if records were historical (from twentieth century); currently most of these species constitute complexes of strikingly similar morphotypes, difficult to separate using optical equipment solely

^*^Type locality in Julebæk Beach, N of Helsingør (Zealand).**Type locality in Frederikshavn (Jutland). *Halobiotus geddesi* nom. inq. is not sufficiently delimited from *H. crispae*

**Fig. 1:**
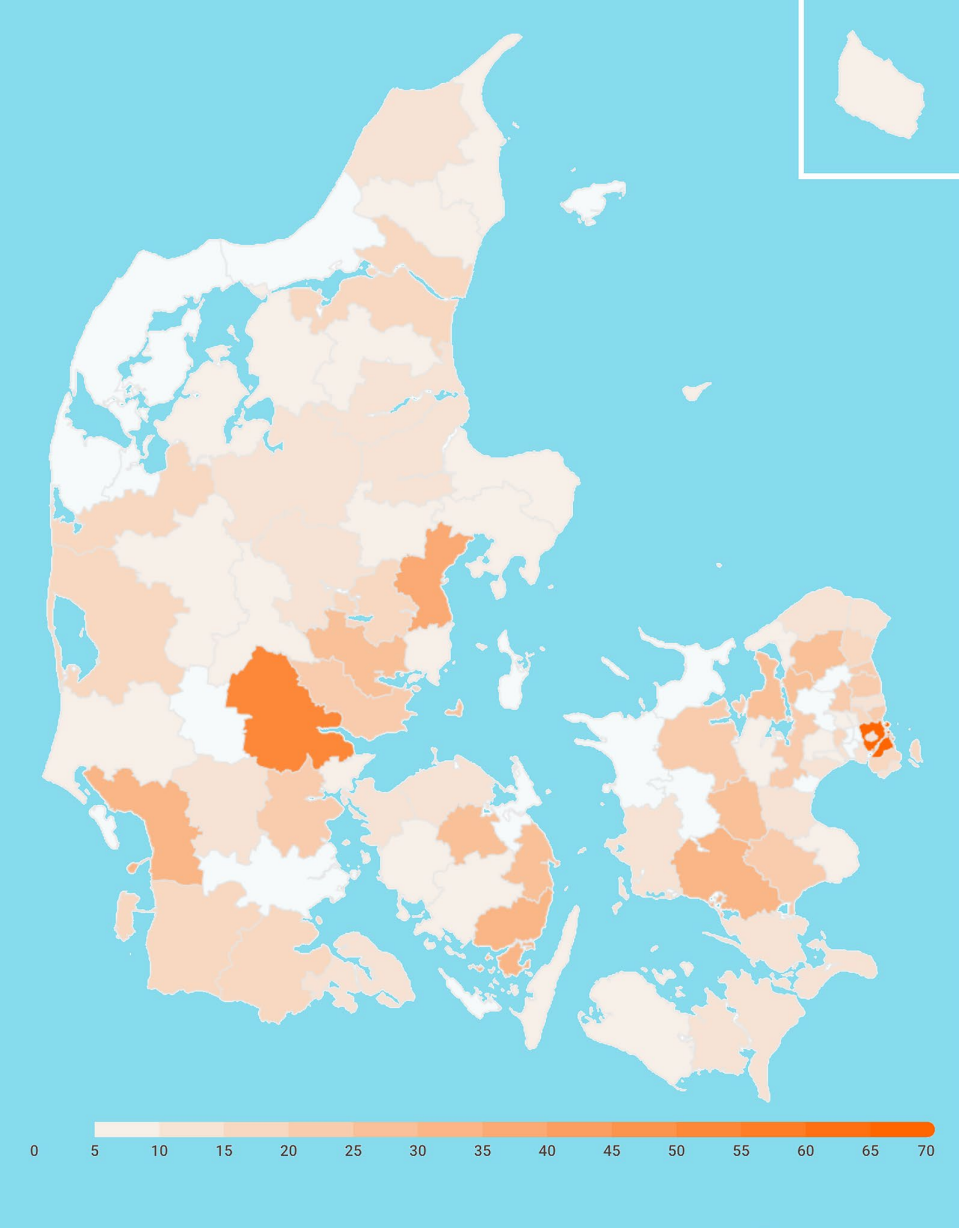
Map depicts the density of schools involved in the Masseeksperiment’23 within all Danish municipalities (inset: Bornholm). The scale refers to the number of schools (each school delivered up to 10 samples)

In order to thoroughly address the fauna of tardigrades dwelling in cryptogams across the country, a citizen science project Masseeksperimentet (https://masseeksperiment.dk/tidligere-eksperimenter/masseeksperiment-2023-mikroliv/, subsequently referred to as ‘Mass Experiment’) was initiated in 2023 in collaboration with the Danish National Center for Science Education, Astra. School classes throughout Denmark (Fig. 1) were involved in collection of cryptogams (bryophytes and lichens) in their respective localities during several weeks in May and early June; pupils also recorded geolocation, habitat, and substrate in the national biodiversity monitoring platform Arter (arter.dk). Samples were later delivered to NHMD and a selected fraction examined using standard laboratory pipeline for tardigrades [17]. In parallel, all cryptogams within these samples were identified by taxonomic specialists, which resulted in a complete database of tardigrades, mosses, liverworts, and lichens. Such approach, in principle, will allow for disclosing any substrate-tardigrade associations, and means that the Mass Experiment is the first mapping of tardigrades and their host cryptogams together throughout an entire country. It is anticipated that integrative taxonomic methodology will greatly facilitate ecological research on tardigrades, a rather sporadically tackled topic up to date [18].

## Methods

### Sampling and sample processing

Around 8.000 samples were collected by school pupils in various regions of Denmark (Fig. 1); cryptogams were packed into small coffee filters, completely dried, if necessary, and sent to NHMD. 676 samples were selected for tardigrade examination based on the amount (typically at least 10 g of dry tissue) and quality (without mould; leprose lichens were discarded) of material and represented the following regions: Zealand 290, Jutland (including Vendsyssel-Thy) 284, Funen 34, Bornholm 24, Amager 13, Lolland 9, Falster 9, Langeland 5, Anholt 4, Samsø 3, Møn 2. The list of all samples with collection data can be found in the Supplementary Material 1. First, all tardigrades were extracted from cryptogams (entire samples were used; the amount of dry substrate varied between 10 and 30 g) as summarised in [17]. In most cases (ca. 90%), entire sediment was poured onto a single Petri dish (⌀ = 10 cm), but when a large amount of soil obscured extraction, it was divided into further 1–2 Petri dishes. Later, cryptogams were analysed and identified at least to genus level (but in more than 80% cases to species level) by specialists, to enable unravelling potential tardigrade-cryptogam associations. This will be addressed in a future paper, entirely devoted to ecological preferences of tardigrades regarding the cryptogam substrate.

### Microscopy and imaging

Specimens for light microscopy were mounted on microscope slides in Hoyer’s medium and secured with cover slips. A brief recapitulation of the procedure can be found in [17]. Permanent slides were analysed in an Olympus BX51 compound microscope with differential interference contrast optics, and in Olympus BX53 microscope associated with a Olympus DP74 digital camera. Slides are deposited in the Jagiellonian University. When required for identification, morphometry was conducted only under BX53. All relevant structures were measured only if their orientation was suitable, without any deformations.

### Morphological primary species hypotheses

**Fig. 2:**
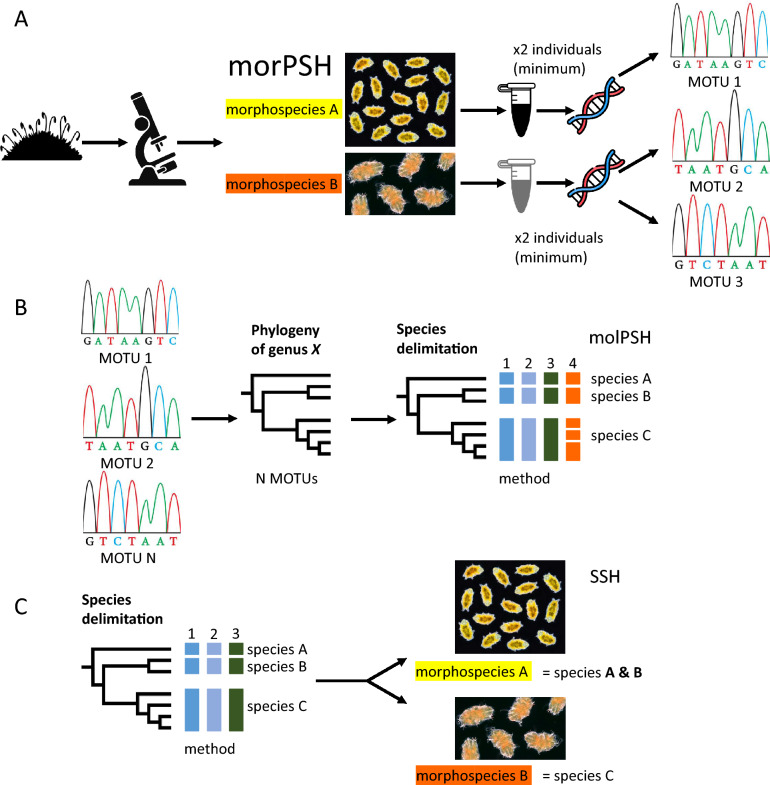
The taxonomic approach applied in the present study: **A** formulation of morphological primary species hypotheses (morPSH) and subsequent DNA barcoding of selected representatives of each morphospecies; **B** molecular operational taxonomic units (MOTUs) used in phylogenetics and molecular species delimitation methods—as a result, molecular primary species hypotheses (molPSH) were posed; **C** integration and cross-validation of both PSHs: the most parsimonious and congruent solutions were sought to restrict the number of secondary species hypotheses (SSH), which mostly corresponded with taxa (see Table 2)

We applied the concept of species hypotheses from Pante et al. [19]. After a quick analysis of morphology in light microscopy, all individuals from a given sample were grouped into morphospecies [20], which constituted morphological primary species hypotheses (Fig. 2A). The following papers, containing trustworthy and most updated information, were used for species delineation in light microscopy: 1. Echiniscidae—[21]; 2. Milnesiidae—[22, 23]; 3. Hypsibiidae—[24–32]; 4. Ramazzottiidae—[33, 34]; 5. Isohypsibiidae—[24, 25, 35]; 6. Macrobiotidae (not identified to species level when eggs were not found)—[7, 36, 37]; 7. Murrayidae—[38].

### Genotyping

Initially, two specimens per each morphospecies from a sample were chosen for DNA barcoding (Fig. 2A); this number was adjusted for populations characterised by atypically wider intraspecific variability (*p*-distance > 3%, morphological deviations, males in the populations of *Milnesium*). DNA was extracted from single tardigrades using Chelex® 100 resin [39, 40]. Hologenophores were recovered after the extraction and mounted on permanent slides in Hoyer’s medium when possible, in other cases, paragenophores were preserved [41]. ITS-2 was used as the basic DNA barcode amplified and sequenced in this survey according to the protocols described in [40]; primers used: Echiniscidae (ITS-3: GCATCGATGAAGAACGCAGC, ITS-4: TCCTCCGCTTATTGATATGC; [42], Eutardigrada (ITS2_Eutar_Ff: CGTAACGTGAATTGCAGGAC, ITS2_Eutar_Rr: TGATATGCTTAAGTTCAGCGG; [33], which also contains specific PCR programme used for all amplifications). In some cases, where additionally ITS-1 and COI could aid in species identification, these markers were sequenced, too. Supplementary Material 2 contains primers and original references for specific PCR programmes in both cases. GenBank accession numbers for sequences obtained in this study are presented in Supplementary Material 3.

### Molecular primary species hypotheses

A final dataset of molecular operational taxonomic units (MOTUs; [43]) was compiled for each genus (Fig. 2A). In many cases, a quick BLAST search [44] allowed for a confident assignment of MOTUs to taxa, chiefly thanks to the influx of recent integrative redescriptions and revisions. Thus, a morphological identification followed by molecular identification converged into a reliable secondary species hypothesis. However, in several other cases (*Milnesium*, *Macrobiotus*, *Ramazzottius*, and *Paramacrobiotus*; the first three genera are the most common taxa in Denmark and frequently co-occur in samples, see below), all MOTUs representing a single genus were used in phylogenetic reconstructions for the purpose of molecular species delimitation [45]. All ITS-2 sequences were aligned with a neotype barcode from *Echiniscus testudo* as outgroup using the ClustalW Multiple Alignment tool [46] implemented and then checked manually in BioEdit ver. 7.2.5 [47]. W-IQ-TREE was used in Maximum Likelihood analyses [48, 49]. Five thousand ultrafast bootstrap (UFBoot) replicates were applied to provide support values for branches [50]. All final consensus trees were visualised by FigTree v.1.4.3 available from https://tree.bio.ed.ac.uk/software/figtree.

Uncorrected pairwise (*p*) distances were calculated in MEGA version 7.0 with a ‘complete deletion’ option [51]. From all suitable delineation methods [45], we chose one distance-based (ASAP; [52]) and one phylogeny-based (bPTP; [53]), with default settings applied to the datasets. That way, we obtained molecular primary species hypotheses (Fig. 2B).

### Data integration and cross-validation

When both molecular and morphological primary species hypotheses were collated, we sought for a maximal congruence between these two sources of evidence. Given that a single universal barcoding gap for all tardigrade lineages is not achievable, at least at present [54], we tended to lump MOTUs more diverging from the remaining MOTUs clearly belonging to the same biological species in cases when both qualitative and quantitative morphology did not indicate any differentiation (see below). This conservative approach might have contributed to a slight underestimation of species richness in *Milnesium* and *Ramazzottius*, but prevented over-splitting of still scarce MOTUs into fictitious species (*e.g*. [55]). In other words, we cross-checked whether molecular primary species hypotheses corresponded with morphospecies, which produced firm secondary species hypotheses (Fig. 2C). The latter can be divided into three groups: (a) named and known taxa; (b) new and unnamed taxa; and (c) taxa, which cannot be reliably identified due to taxonomic obscurities (Table 2).

**Table 2:** List of Danish cryptogam-dwelling Tardigrada. Asterisk (*) signifies that a species was identified only via morphology (*E. testudo* has already been cross-validated by molecular data, thus not marked)

Family	Species and authority	Remarks
Echiniscidae	1. *Echiniscus blumi* Richters, 1903	Widespread in Denmark, but rare and not numerous
	2. *Echiniscus merokensis* Richters, 1904	Restricted to Jutland, rare; one population contained males, which is the first record of a bisexual population in this species
	3. *Echiniscus quadrispinosus* Richters, 1902	Restricted to Jutland, rare
	4. *Echiniscus testudo* (Doyère, 1840)	Found in Zealand, Amager, Langeland, and Bornholm, rare and not numerous
Milnesiidae	5. *Milnesium berladnicorum* Ciobanu et al., 2014	Rare; reliable reports from the Palaearctic and Afrotropics [8]. Males absent
	6. *Milnesium dornensis* Ciobanu et al., 2015	Relatively widespread and common in Denmark; probably Palaearctic. Males present
	7. *Milnesium pseudotardigradum* Surmacz et al., 2019	Restricted to Zealand, but might have been overlooked due to the fact that not all *Milnesium* populations were barcoded and this species is extremely difficult to distinguish from *M. tardigradum* [23] when a few individuals are available; likely cold stenothermic [8]. Males absent
	8. *Milnesium tardigradum* Doyère, 1840	Relatively widespread and common in Denmark; reliable reports from the Palaearctic and Afrotropics [8, 82, 83]. Males absent
	9. *Milnesium variefidum* Morek et al., 2016	Widespread in Denmark, rare; probably cold stenothermic and Palaearctic [8]. Males absent
	10. *Milnesium* sp. nov. 1	The most common and widespread of all Danish *Milnesium* spp.; claw configuration [2-3]–[3-2], broad buccal tube, pseudoplates present, males present. Not detected in the survey of Morek et al. [8]. Description in preparation
	11. *Milnesium* sp. nov. 2	Widespread in Denmark, but rare and not numerous; claw configuration [2-3]–[2-2], narrow buccal tube, pseudoplates present, males absent. Represents species #5 (populations PT.010A + 059) from [8]
	12. *Milnesium* sp. nov. 3	Found only in two localities on Jutland and Zealand; claw configuration [3-3]–[3-3], narrow buccal tube, pseudoplates present, males absent. Represents species #9 (population GL.055) from [8]
	13. *Milnesium* sp. nov. 4	Fund only in one locality on Jutland; claw configuration [2-3]–[3-3], broad buccal tube, pseudoplates present, males absent. Not detected in the survey of Morek et al. [8]
Hypsibiidae	14. *Adropion scoticum* (Murray, 1905)	Restricted to Jutland, rare. Verified according to the recent redescription [30]
	15. *Astatumen* sp. 1	Small species (typically < 200 μm), internal bars II–III present. Conspecific with *Astatumen* sp. nov. 1 from [31]
	16. *Astatumen* sp. 2	Large species (adults > 400 μm), internal bars II–III not always visible. Belongs to the clade *Astatumen bartosi* + *Astatumen* aff. *trinacriae* 2 (Italy) & 3 (Hungary) from [31]. Impossible to tell whether the species is new due to dated descriptions of *A. bartosi* and *A. trinacriae*
	17. *Diphascon pingue* (Marcus, 1936)	Widespread in Denmark, but rare and not numerous
	18. *Guidettion prorsirostre* (Thulin, 1928)*	Found only in one locality on Zealand. Verified according to the recent redescription [30]
	19. *Hypsibius* cf. *convergens* (Urbanowicz, 1925)*	Widespread in Denmark, but rare and not numerous. A confident identification is not possible because of the dated description and the presence of a pseudocryptic species complex
	20. *Hypsibius dujardini* (Doyère, 1840)	Widespread and common in Denmark. Verified according to the redescription [29]
	21. *Hypsibius pallidus* Thulin, 1911*	Found only in three localities on Jutland and Zealand
	22. *Hypsibius scabropygus* Cuénot, 1929	Widespread and common in Denmark
	23. *Hypsibius* sp. nov	Found only in one locality on Jutland; closely related with *H. scabropygus*
	24. *Mesocrista revelata* Gąsiorek et al., 2016	Found only in two localities on Jutland
	25. *Notahypsibius pallidoides* (Pilato et al., 2011)	Relatively widespread and common in Denmark
	26. *Pilatobius bullatus* (Murray, 1905)	Found only in one locality on Zealand, but another record comes from Jutland [67]
	27. *Pilatobius* cf. *rugosus* (Bartoš, 1935)	Found only in one locality on Zealand. Verified according to the recent diagnosis [30]
	28. *Platicrista angustata* (Murray, 1905)	Found only in two localities on Jutland. Verified according to the recent redescription [30]
Ramazzottiidae	29. *Ramazzottius kretschmanni* Guidetti et al., 2022	Found only in one locality on Zealand. First record outside Germany [34]
	30. *Ramazzottius oberhaeuseri* (Doyère, 1840)	Found only on Zealand, Amager, and Bornholm; not numerous. Verified according to the recent redescription [33]
	31. *Ramazzottius* sp. nov. 1	Represents species #1 (populations from Germany, Switzerland, Poland, and Sweden) from [33]. Widespread and common in Denmark
	32. *Ramazzottius* sp. nov. 2	Represents species #7 (population from Portugal) from [33]. Widespread and common in Denmark
	33. *Ramazzottius* sp. nov. 3	Found only on Zealand and Bornholm; not numerous
Isohypsibiidae	34. *Eremobiotus ginevrae* Lisi et al., 2016*	Found only in one locality on Langeland
	35. *Isohypsibius* cf. *prosostomus* Thulin, 1928*	Widespread in Denmark, but rare and not numerous. A confident identification is not possible because of the dated description
	36. *Ursulinius* cf. *lunulatus* (Iharos, 1966)	Found only in two localities on Zealand. A confident identification is not possible because of the dated description and the lack of data on intraspecific variability in development of dorsal gibbosities in *Ursulinius*
	37. *Ursulinius* cf. *pappi* (Iharos, 1966)	Found only in one locality on Jutland. See above for identification
Macrobiotidae	38. *Macrobiotus hannae* Nowak & Stec, 2018	Found only in three localities on Jutland and Zealand
	39. *Macrobiotus hufelandi* C.A.S. Schultze, 1834	Relatively widespread and common in Denmark
	40. *Macrobiotus macrocalix* Bertolani & Rebecchi, 1993	Relatively widespread and common in Denmark
	41. *Macrobiotus polonicus* Pilato et al., 2003	Relatively widespread and common in Denmark
	42. *Macrobiotus* cf. *polonicus* Pilato et al., 2003	Relatively widespread and common in Denmark. Corresponds with Swedish populations of *M*. cf. *polonicus* from [70]
	43. *Macrobiotus scoticus* Stec et al., 2017	Widespread and common in Denmark
	44. *Macrobiotus sottilei* Pilato et al., 2012	Widespread and common in Denmark
	45. *Macrobiotus vladimiri* Bertolani et al., 2011	Widespread and common in Denmark
	46. *Mesobiotus mandalori* Erdmann et al., 2024	Widespread in Denmark, but rare and not numerous. First record outside Poland [84]
	47. *Mesobiotus* sp. 1	Yellow species with many multi-shaped pores. Relatively widespread and common in Denmark
	48. *Mesobiotus* sp. 2	White/transparent species with a few round pores. Widespread in Denmark, but rare and not numerous
	49. *Mesobiotus* sp. 3	White/transparent species with a few round pores with dark rugged edges. Widespread in Denmark, but rare and not numerous
	50. *Minibiotus* sp. 1	Found only in one locality on Jutland. Three macroplacoids and microplacoid in the pharynx; aporous cuticle and tiny granulation present on legs IV
	51. *Paramacrobiotus fairbanksi* Schill et al., 2010	Widespread in Denmark, but rare and not numerous. Cosmopolitan [7, 85]
	52. *Paramacrobiotus richtersi* (Murray, 1911)	Found only in three localities on Zealand and Fyn. Verified according to the redescription [7]
	53. *Paramacrobiotus* sp. 1	Found only in three localities on Jutland and Fyn. Belongs in the *richtersi* group [7]
	54. *Tenuibiotus* sp. 1*	Found only in one locality on Zealand. This finding reveals the presence of the genus *Tenuibiotus* in Denmark, not detected before [16], but the lack of eggs and more individuals for DNA barcoding prevented species identification
Murrayidae	55. *Paramurrayon meieri* Guidetti et al., 2022	First record outside Norway [38, 57]

## Results

**Fig. 3:**
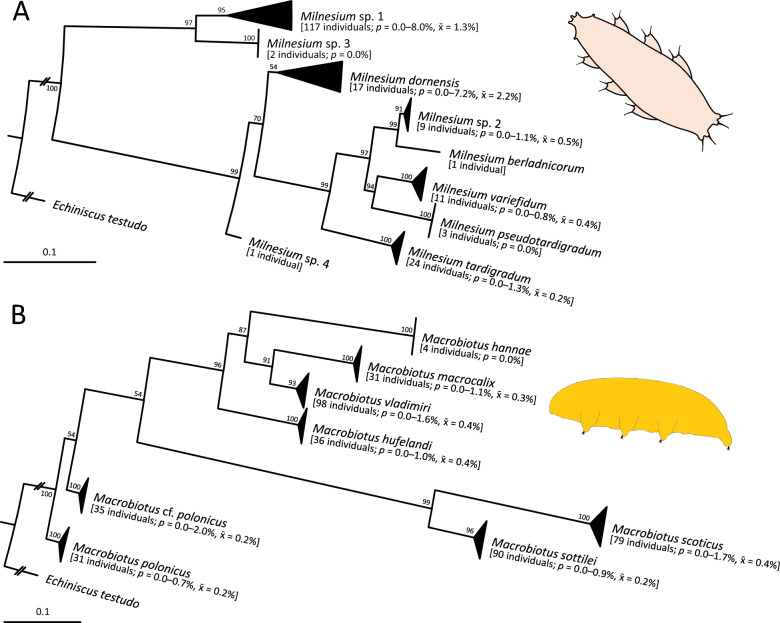
Integrated SSH for the genera: **A** *Milnesium*; **B** *Macrobiotus*, as two examples of conducted analyses. Maximum Likelihood trees were rooted on an outgroup species *Echiniscus testudo*; scale bars represent substitutions per position. *p* signifies ranges of uncorrected pairwise distances

Out of 676 examined samples, 171 (25%) were without tardigrades (Supplementary Material 1). The remaining 505 samples (75%) contained tardigrades representing seven families, 21 genera, and 55 species. At least nine spp. (16%) are new to science (Table 2). Heterotardigrades were represented only by a single family Echiniscidae and four widespread spp. of *Echiniscus*. Apochelan eutardigrades were split into nine *Milnesium* spp., of which *Milnesium* sp. nov. 1 apparently is the most common species of the genus in Denmark (63% of all sequenced individuals; Fig. 3A); notable is the presence of two singletons (*M. berladnicorum*, *M*. sp. nov. 4), followed by two other rare spp. (*M. pseudotardigradum*, *M*. sp. nov. 3). For two dioecious *Milnesium* spp. (*M. dornensis*, *M*. sp. nov. 1), the range of molecular distances was up to 8% because several specimens (< 5% of all sequenced individuals) greatly increased the intraspecific variability (Supplementary Material 4) in ITS-2. This was not accompanied by any easily noticeable morphological differences between studied populations, and was not treated as a sign of interspecific divergence.

Parachelan eutardigrades were classified within five families, of which one—Murrayidae (*Paramurrayon meieri*)—was present only in one locality. The second rarest family was the Isohypsibiidae, scarcely represented by four spp. As predicted, the most common families were Hypsibiidae (15 spp., including truly ubiquitous *Hypsibius dujardini* and *H. scabropygus*), Ramazzottiidae (five spp., with the most common species: *Ramazzottius* sp. nov. 1 and 2), and Macrobiotidae (17 spp., including seven common *Macrobiotus* spp.). In contrast to *Milnesium*, none of the spp. exhibited intraspecific *p* > 2% (Fig. 3B, Supplementary Material 5), which is lower than usually accepted 3% DNA barcoding threshold in molecular species delineation studies [56].

## Discussion

### Danish fauna

At first, we compare our results with the historical records (Table 1) and the extensive soil eDNA survey [16], which dealt only with Danish tardigrade fauna. Then, we expand our comparisons to the Norwegian fauna, which has been recently addressed in a great detail using traditional approach based on light microscopy identification [57], aiming at pinpointing taxa not disclosed in Denmark, but probably present in the country.

We found six out of 11 families reported by Pust et al. [16], enriched with the presence of heterotardigrade Echiniscidae, which do not inhabit soil (alternatively, heterotardigrades may require specific primers to be revealed in an eDNA dataset due to large insertions in V4 region of 18S rRNA; [16, 58]). Three out of five families absent in our dataset (eohypsibiids, microhypsibiids, adorybiotids) are generally found sporadically and the first two seem to exhibit preferences towards leaf litter (*Bertolanius*), soil (*Microhypsibius*), and even water bodies (*Eohypsibius*, *Microhypsibius*) or springs [59, 60]. Hexapodibiids are soil-dwelling [61], and most doryphoribiids (*Grevenius*, *Thulinius*) reported by Pust et al. [16] are limnic [62]. Therefore, the absence of all five lineages in our samples was not unexpected.

**Fig. 4:**
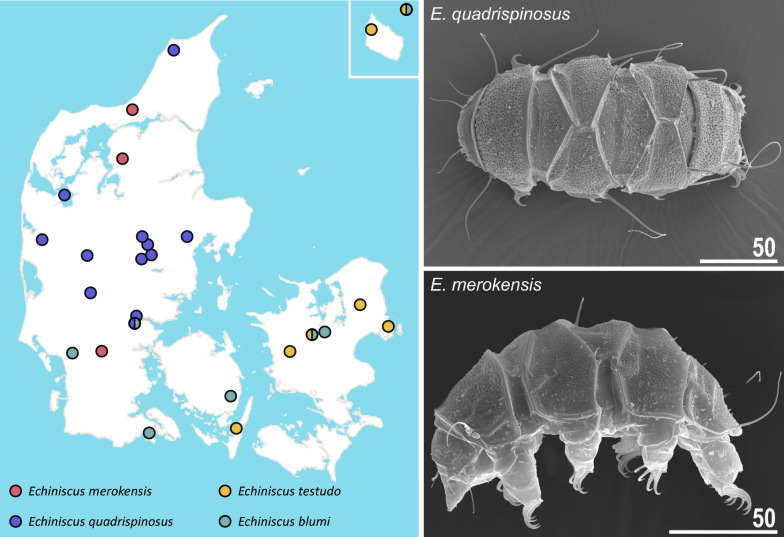
An example of biogeographic regionalisation within the Danish fauna: genus *Echiniscus*. Scale bars in micrometres

*Echiniscus* showed an interesting regionalisation: only *E. blumi* is widespread in Denmark, whereas *E. testudo* is present on islands east of Jutland. In contrast, *E. merokensis* and *E. quadrispinosus* (Fig. 4) are present only in Jutland. *Echiniscus granulatus*, typical for mosses from carbonate bedrock [21, 25], was not found (isolated, potentially promising localities on Møn and Bornholm did not yield any record). An unidentified *Echiniscus* and *E. arctomys* sp. inq. were reported from Bornholm [63], but these records must remain unverifiable due to the destruction of the European-originating part of the Richters collection (H. Dastych, pers. observation) and may represent an aberrant form of any of the four spp. reported herein (*E. merokensis* and *E. blumi-canadensis* complex are known for large morphological variability; [21, 64]) or a *Pseudechiniscus* species as well. Hallas [65] reported a member of the genus *Pseudechiniscus* (unlikely to represent *P. suillus s.s*.) from a suitable habitat on the rocks of Helligdommen (NE Bornholm). However, our resampling of this locality did not unravel the presence of any echiniscid. In general, no other echiniscids were anticipated to be present in Denmark.

The number of Danish *Milnesium* spp. increased from one (*M. tardigradum* positively verified) to nine, including five described and further three previously characterised genetically in a large-scale survey [8]. Only *M*. sp. nov. 4 has not been sequenced previously, which demonstrates that even in relatively well-sampled biogeographic regions, such as the Palaearctic, so far undescribed spp. can be found. Moreover, this increment in known biodiversity plainly corroborates the argumentation of Ugarte & Garraffoni [66], who argued that most historical tardigrade distribution records are not usable for modern taxonomic and ecological research purposes since they can represent multiple, even unrelated spp. Our records, associated with a basic DNA barcode, allow for direct species comparisons and thus can be coupled with future faunistic data for tardigrades more easily.

Among hypsibiids, we confirmed the presence of *A. scoticum*, *H. dujardini*, and *P. bullatus* in Denmark. It is likely that *D. alpinum* sp. dub. reported by Hallas & Yeates [67] represents in fact *D. pingue* as the two spp. share a long history of taxonomic confusion [68]. We did not find either *Mixibius* cf. *saracenus*, a rare and primarily aquatic species revealed by Pust et al. [16], or *D. oculatum*, a rare species dwelling mainly in mountains [25]. Ramazzottiids are represented only by *Ramazzottius*, which instead is among top-three most common genera and embraces at least five distinct spp. *Ramazzottius oberhaeuseri* and *R. kretschmanni*, two named spp., are actually much rarer than two undescribed spp. (*R*. sp. nov. 1 and 2) previously characterised genetically [33]. Among isohypsibiids, we did not find *Dianea* cf. *sattleri* reported by Pust et al. [16], and the genus *Dianea* should be present in Denmark as it is present in the neighbouring Sweden [12] and Germany [69]. All four isohypsibiids are rare and elusive (Table 2).

Macrobiotids are the most speciose family, and the most interesting finding is the disclosure of the presence of *Tenuibiotus* in Denmark. A single population of *Minibiotus* from Jutland does not represent *M. intermedius* (the neotypic COI barcode ON005160 of *M. intermedius* does not match the Danish population) and its taxonomic status (a new similar species or previously described species lacking DNA barcodes) is uncertain. Both *Macrobiotus* and *Mesobiotus* are much commoner than *Paramacrobiotus*. *Macrobiotus hufelandi* is confirmed as an element of Danish fauna [65]. In total, nine out of 14 valid spp. reported by Hallas [65] and Hallas & Yeates [67] were positively verified.

As could have been assumed, the Norwegian fauna is more diverse, encompassing almost three times more spp. than Danish fauna [15, 57]. This fact is glaring when *e.g*. the number of echiniscid taxa is compared (seven genera, 31 spp. vs one genus, four spp.; although it should be noted that Guidetti et al. [57] seem to greatly overestimate the number of *Echiniscus* spp., probably due to large intraspecific variability interpreted as interspecific disparities). The presence of several recently researched spp. (*P. meieri*, [38]; *Microhypsibius*, [57]) or species groups (*Macrobiotus persimilis-polonicus* complex, [70]) in Denmark, Norway, and Sweden suggests similarity of faunae and biogeographic structuring [9]. With all genera recorded by Pust et al. [16] and by us, a direct comparison can be made with the checklist from [57]. Only one genus, *Itaquascon*, which is otherwise extremely rare, could be additionally present in Denmark. Apart from it, in our opinion the present contributions revealed a large fraction of cryptogam-inhabiting genera (among aquatic genera not caught by Pust et al. [16], surely at least one *Dactylobiotus* species is present in Denmark, but its taxonomic affinity is dubious, see Table 1), and a special attention should be given to naming new species in the next step.

### Citizen science and faunistics

Reaching the scope of our research would not be possible without an immense effort of pupil and teacher helpers. This is another example of how beneficial the participation of local communities can be in the case of biodiversity research. Similar projects were concluded with a great advancement of knowledge on life history of seahorses [71], monitoring of invasive species [72], conservation biology [73], or species discovery [74]. Given how undersampled vast areas of the globe are in terms of tardigrade diversity, the involvement of citizen sample collectors creates a favourable perspective for efficient formation of taxonomic checklists. Our paper presents the first integrative checklist of tardigrades of an entire country, quadrupling the number of Danish water bears.

### Conclusions

Limno-terrestrial, cryptogam-dwelling tardigrade fauna of Denmark is typically Palaearctic, with some cosmopolitan elements (*E. testudo*, *P. fairbanksi*). Species α-diversity varies from low to moderate, depending on the family, but it is expected to increase providing that limnic habitats (ponds, lakes, bogs, and rivers) will be sampled. Despite this, a significant fraction of new undescribed spp. warrants next biodiversity surveys and future taxonomic work, preferably drawing from multiple lines of evidence [7, 21, 32, 75].

## Supplementary Information

Supplementary Material 1: S1. Collection data. Quantity means the total approximate number of all tardigrades found in a sample. The list of tardigrade records per sample is given in the form of three-letter abbreviations formed ad hoc from the first three letters of the generic and specific name, e.g. ‘Ech.tes’ signifies *Echiniscus testudo*. Each record is followed by an approximate number of specimens, categorised into these groups: 1, <10 (2–9), <50 (10–49), <100 (50–99), ≥100, extracted from a single Petri dish (PD).

Supplementary Material 2: S2. Primers and PCR programmes for ITS-1 and COI markers, sequenced additionally in this study.

Supplementary Material 3: S3. GenBank accession numbers.

Supplementary Material 4: S4. Matrices of intraspecific *p*-distances for *Milnesium*.

Supplementary Material 5: S5. Matrices of intraspecific *p*-distances for *Macrobiotus*.

## Data Availability

All data is published in the manuscript and its supplementary materials. Sequences are deposited in GenBank.
