# Supplementary material for: Massive citizen science sampling and integrated taxonomic approach unravel Danish cryptogam-dwelling tardigrade fauna
Source: Front Zool. 2024 Oct 21;21:27. doi: 10.1186/s12983-024-00547-x (PMC11492576; doi:10.1186/s12983-024-00547-x)
Supplement: Supplementary file 2 — Supplementary Material 2: S2. Primers and PCR programmes for ITS-1 and COI markers, sequenced additionally in this study. [file 12983_2024_547_MOESM2_ESM.docx]

**SM.2.** Primers and references for specific protocols for amplification of ITS-1 and COI fragments sequenced in the study.

| **DNA fragment** | **Primer name** | **Primer direction** | **Primer sequence (5’-3’)** | **Primer source** | **PCR programme*** |
| --- | --- | --- | --- | --- | --- |
| **ITS-1** | ITS1_Echi_F | forward | CCGTCGCTACTACCGATTGG | Gąsiorek et al. (2019) | Wełnicz et al. (2011) |
|  | ITS1_Echi_R | reverse | GTTCAGAAAACCCTGCAATTCACG |  |  |
| **COI** | bcdF01 | forward | CATTTTCHACTAAYCATAARGATATTGG | Dabert et al. (2008) |  |
|  | bcdR04 | reverse | TATAAACYTCDGGATGNCCAAAAAA |  |  |
|  | LCO1490_JJ | forward | CHACWAAYCATAAAGATATYGG | Astrin & Stüben (2008) |  |
|  | HCO2198_JJ | reverse | AWACTTCVGGRTGVCCAAARAATCA |  |  |

* – All PCR programmes are also provided in Stec et al. (2020).

Astrin J.J. & Stüben P.E. (2008) Phylogeny in cryptic weevils: molecules, morphology and new genera of western Palaearctic Cryptorhynchinae (Coleoptera : Curculionidae). Invertebrate Systematics 22: 503–522. https://doi.org/10.1071/IS07057

Dabert J., Ehrnsberger R. & Dabert M. (2008) *Glaucalges tytonis* sp. nov. (Analgoidea: Xolalgidae) from the barn owl *Tyto alba* (Strigiformes: Tytonidae): compiling morphology with DNA barcode data for taxa descriptions in mites (Acari). Zootaxa 1719: 41–52. <https://doi.org/10.11646/zootaxa.1719.1.2>

Gąsiorek P., Jackson K.J., Meyer H.A., Zając K., Nelson D.R., Kristensen R.M. & Michalczyk Ł. (2019) *Echiniscus virginicus* complex: the first case of pseudocryptic allopatry and pantropical distribution in tardigrades. Biological Journal of the Linnean Society 128: 789–805. <https://doi.org/10.1093/biolinnean/blz147>

Stec D., Kristensen R.M. & Michalczyk Ł. (2020) An integrative description of *Minibiotus ioculator* sp. nov. from the Republic of South Africa with notes on *Minibiotus pentannulatus* Londoño et al., 2017 (Tardigrada: Macrobiotidae). Zoologischer Anzeiger 286: 117–134. <https://doi.org/10.1016/j.jcz.2020.03.007>

Wełnicz W., Grohme M.A., Kaczmarek Ł., Schill R.O. & Frohme M. (2011) ITS-2 and18S rRNA data from *Macrobiotus polonicus* and *Milnesium tardigradum* (Eutardigrada, Tardigrada). Journal of Zoological Systematics and Evolutionary Research 49: 34–39. <https://doi.org/10.1111/j.1439-0469.2010.00595.x>
