# Supplementary material for: Massive citizen science sampling and integrated taxonomic approach unravel Danish cryptogam-dwelling tardigrade fauna
Source: Front Zool. 2024 Oct 21;21:27. doi: 10.1186/s12983-024-00547-x (PMC11492576; doi:10.1186/s12983-024-00547-x)
Supplement: Supplementary file 3 — Supplementary Material 3: S3. GenBank accession numbers. [file 12983_2024_547_MOESM3_ESM.docx]

**SM.3.** List of GenBank accession numbers.

| **Family** | **Species and authority** | **ITS-2** | **ITS-1** | **COI** |
| --- | --- | --- | --- | --- |
| Echiniscidae | 1. *Echiniscus blumi* | PQ354426-8 | PQ350065-7 | PQ356801-3 |
|  | 1. *Echiniscus merokensis* | PQ354429-38 | PQ350068-77 | PQ356804-11 |
|  | 1. *Echiniscus quadrispinosus* | PQ354439-47 | PQ350078-86 | PQ356812-20 |
|  | 1. *Echiniscus testudo* | – | – | – |
| Milnesiidae | 1. *Milnesium berladnicorum* | PQ354448 | – | – |
|  | 1. *Milnesium dornensis* | PQ354449-65 | – | – |
|  | 1. *Milnesium pseudotardigradum* | PQ354466-8 | – | – |
|  | 1. *Milnesium tardigradum* | PQ354469-92 | – | PQ356821-2 |
|  | 1. *Milnesium variefidum* | PQ354493-503 | – | – |
|  | 1. *Milnesium* sp. nov. 1 | PQ354504-622 | – | PQ356823-9 |
|  | 1. *Milnesium* sp. nov. 2 | PQ354623-31 | – | PQ356830-1 |
|  | 1. *Milnesium* sp. nov. 3 | PQ354632-3 | – | PQ356832 |
|  | 1. *Milnesium* sp. nov. 4 | PQ354634 | – | – |
| Hypsibiidae | 1. *Adropion scoticum* | PQ354635-9 | – | – |
|  | 1. *Astatumen* sp. 1 | PQ354640-1 | – | – |
|  | 1. *Astatumen* sp. 2 | PQ354642-4 | – | – |
|  | 1. *Diphascon pingue* | PQ354645 | – | – |
|  | 1. *Guidettion prorsirostre* | – | – | – |
|  | 1. *Hypsibius* cf. *convergens* | – | – | – |
|  | 1. *Hypsibius dujardini* | PQ354646-77 | – | PQ356833-6 |
|  | 1. *Hypsibius pallidus* | – | – | – |
|  | 1. *Hypsibius scabropygus* | PQ354678-95 | – | PQ356837-61 |
|  | 1. *Hypsibius* sp. nov. | PQ354696 | – | PQ356862 |
|  | 1. *Mesocrista revelata* | PQ354697-9 | – | – |
|  | 1. *Notahypsibius pallidoides* | PQ354700-3 | – | – |
|  | 1. *Pilatobius bullatus* | PQ354704-5 | – | PQ356863-4 |
|  | 1. *Pilatobius* cf. *rugosus* | – | – | PQ356865 |
|  | 1. *Platicrista angustata* | PQ354706-8 | – | – |
| Ramazzottiidae | 1. *Ramazzottius kretschmanni* | PQ354709 | – | – |
|  | 1. *Ramazzottius oberhaeuseri* | PQ354710-3 | – | PQ356866-8 |
|  | 1. *Ramazzottius* sp. nov. 1 | PQ354714-851 | – | PQ356869-78 |
|  | 1. *Ramazzottius* sp. nov. 2 | PQ354852-932 | – | PQ356879-92 |
|  | 1. *Ramazzottius* sp. nov. 3 | PQ354933-42 | – | – |
| Isohypsibiidae | 1. *Eremobiotus ginevrae* | – | – | – |
|  | 1. *Isohypsibius* cf. *prosostomus* | – | – | – |
|  | 1. *Ursulinius* cf. *lunulatus* | PQ354943-6 | – | – |
|  | 1. *Ursulinius* cf. *pappi* | PQ354947-50 | – | – |
| Macrobiotidae | 1. *Macrobiotus hannae* | PQ354951-4 | – | – |
|  | 1. *Macrobiotus hufelandi* | PQ354955-90 | – | – |
|  | 1. *Macrobiotus macrocalix* | PQ354991-5021 | – | – |
|  | 1. *Macrobiotus polonicus* | PQ355022-52 | – | – |
|  | 1. *Macrobiotus* cf. *polonicus* | PQ355053-87 | – | – |
|  | 1. *Macrobiotus scoticus* | PQ355088-166 | – | – |
|  | 1. *Macrobiotus sottilei* | PQ355167-256 | – | – |
|  | 1. *Macrobiotus vladimiri* | PQ355257-354 | – | – |
|  | 1. *Mesobiotus mandalori* | PQ355355-67 | – | – |
|  | 1. *Mesobiotus* sp. 1 | PQ355368-401 | – | – |
|  | 1. *Mesobiotus* sp. 2 | PQ355402-8 | – | – |
|  | 1. *Mesobiotus* sp. 3 | PQ355409-11 | – | – |
|  | 1. *Minibiotus* sp. 1 | PQ355412 | – | PQ356893 |
|  | 1. *Paramacrobiotus fairbanksi* | PQ355413-21 | – | – |
|  | 1. *Paramacrobiotus richtersi* | PQ355422 | – | – |
|  | 1. *Paramacrobiotus* sp. 1 | PQ355423-4 | – | – |
|  | 1. *Tenuibiotus* sp. 1 | – | – | – |
| Murrayidae | 1. *Paramurrayon meieri* | PQ355425-6 | – | – |
